# The Localisation of a Tracheosophageal Shunt during Laryn(-gopharyn)gectomy Determines the Risk of Shunt Insufficiency

**DOI:** 10.3390/jcm12247628

**Published:** 2023-12-11

**Authors:** Felix Johnson, Andreas Knopf

**Affiliations:** 1 Department of Otorhinolaryngology, Head and Neck Surgery, Technical University of Munich, 80333 Munich, Germany; felix.johnson@i-med.ac.at; 2 Department of Otorhinolaryngology, Head and Neck Surgery, Faculty of Medicine, University of Innsbruck, 6020 Innsbruck, Austria; 3 Department of Otorhinolaryngology, Head and Neck Surgery, Faculty of Medicine, University of Freiburg, 79085 Freiburg im Breisgau, Germany

**Keywords:** shunt insufficiency, voice prosthesis, laryngectomy, complication, laryngeal cancer, fistula

## Abstract

Background: Tracheoesophageal shunt insufficiency (TESI) is a common and potentially life-threatening complication after laryn(-gopharyn)gectomy (L(P)E). We investigated whether TESI could be the result of a specific shunt location. Methods: A monocentric, retrospective cohort analysis of 171 consecutively treated L(P)E patients was performed. Patients with a secondary prosthesis instillation and patients with insufficient postoperative imaging were excluded. Disease related data as well as location of primary voice prosthesis were assessed. Results: The cohort was divided into 62 TESI-positive and 109 TESI-negative individuals. The mean time from surgery to TESI was 32 months. No differences were observed in gender, age, tumor localization, T/R/M-status. Surgery without adjuvant therapy was more often performed in TESI-negative individuals when compared with their positive counterparts. However, Cox regression including T/N status, therapy and categorized distance of the tracheoesophageal shunt to the manubrium (≤1.5 cm vs. >1.5 cm) revealed that a distance of ≤1.5 cm was associated with a 2.1-fold increased risk of TESI, while all other parameters did not influence the event-free survival. Conclusions: Primary shunt positioning ≤1.5 cm to the ridge of the manubrium is associated with an increased risk of TESI. In these individuals secondary shunt operation resulting in a position >1.5 cm distant to the manubrium should be recommended.

## 1. Introduction

Laryn(-gopharyn)gectomy (L(P)E) represents, beside primary conservative approaches, a therapeutic option in locally advanced cancers of the larynx and hypopharynx. Since the first laryngectomy was performed in 1873 by Billroth, the complete removal of the larynx with concurrent tracheocutaneous anastomosis has been standardized. In 2021, approximately 1011 laryngectomies were performed in Germany [1]. Head and neck squamous cell cancers are, with an annual incidence of 660,000 cases and an estimated 325,000 deaths, one of the most common malignancies worldwide [2,3]. However, the loss of patients’ own voice is one of the factors that is most highly deplored by patients following L(P)E, and has a large impact on overall quality of life. While the electrolarynx and esophageal voice were utilized to overcome the functional loss, but both of these a-laryngeal voices are of poor quality. A large percentage of L(P)E patients receive either a primary or secondary tracheoesophageal shunt with the intent of embedding a voice prosthesis as this has consistently been shown to produce superior speech quality, speech intelligibility, ease of use, and a higher quality of life [4,5]. The tracheoesophageal shunt is a surgically created fistula connecting the trachea with the neopharynx. The shunt is occupied with a voice prosthesis, which allows air to enter the esophagus but prevents aspiration of fluids or solids. With the advent of the tracheoesophageal shunt patients could produce a high quality esophageal voice without having to swallow air and then immediately and forcibly expel air [6]. As a result, the tracheoesophageal shunt with voice prosthesis has become the gold standard for voice rehabilitation in L(P)E patients, which is an important factor towards improving quality of life [7,8].

However, the tracheoesophageal shunt has considerable drawbacks. The iatrogenically produced shunt must contain a voice prosthesis, which is comprised of foreign material. Both the voice prosthesis itself as well as the shunt can misfunction. Tracheoesophageal shunt insufficiency (TESI) is a common and often reccurring problem for patients who have received L(P)E [9,10]. Additionally, TESI can lead to serious, life-threatening complications, such as aspiration, pneumonia, sepsis, mediastinitis, cervical cellulitis, or other infections [11,12,13]. We hypothesized that a caudal positioning of the voice prosthesis is associated with an increased risk of TESI by mechanical alteration, e.g., by changing the tracheostomy tube.

The purpose of this study is to investigate whether TESI could be the result of a caudal shunt positioning during shunt surgery.

## 2. Materials and Methods

We conducted a monocentric, retrospective cohort analysis that included all patients who consecutively underwent L(P)E between 2004–2014. Patients who received tracheoesophageal shunt operation and voice prosthesis during L(P)E were included (Provox I^®^, Atos Medical, Troisdorf, Germany). The hospital records of these patients were analyzed for the subsequent necessity of in-hospital treatment for TESI.

Patients with a secondary tracheoesophageal shunt operation after L(P)E, patients who never received a barium swallow and/or postoperative imaging as well as patients with isolated prosthesis malfunction were excluded.

**Figure 1:**
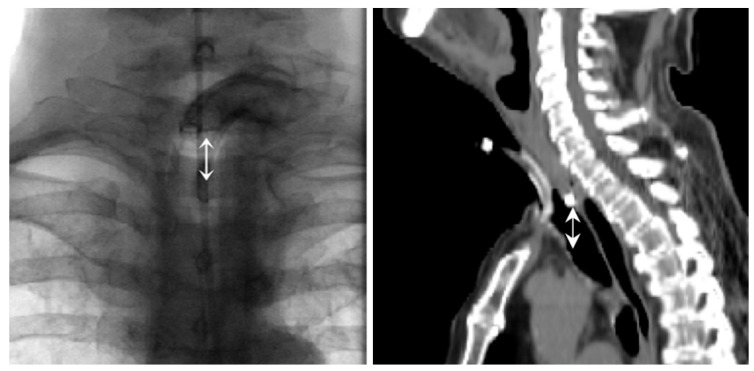
Barium swallow and CT scan (sagittal plane). Distance between lower voice prosthesis margin and manubrium ridge.

We used the university patient digital database SAP to determine when patients were admitted to the ENT hospital ward for TESI. Further information, such as the treatment undertaken, treatment success rate, and tumor related data were documented. In order to determine the anatomical localization of the tracheoesophageal shunt, we utilized calibrated post-operative CT imaging or barium swallows seven to 10 days after L(P)E. The distance from the lower ridge of the manubrium sterni to the lower edge of the tracheoesophageal shunt was measured using the radiological viewing program SECTRA. Assessment of the radiopaque voice-prosthesis and manubrium sterni allowed a reliable analysis of shunt positioning in all patients and avoid soft tissue overlap (Figure 1). Cut-off was analyzed by repeater-operator-characteristic (ROC-analysis) and subsequent Youden-Test. Subsequently, the entire cohort was and categorized in two groups (≤1.5 cm vs. >1.5 cm).

Differences between the groups were analyzed using the Chi square test and Fisher exact test for categorical, and the unpaired student’s t-test for continuous variables. Event-free survival (OS) was assessed measuring the time from treatment to the first occurrence of hospital required TESI treatment. Event rates and curves were calculated and illustrated by the Kaplan–Meier method and further analyzed by the log-rank test. Variables that revealed prognostic or effect modifying potential on the outcome were subsequently evaluated by the proportional Cox regression for forward selection. *p*-values <0.05 were considered statistically significant. Statistical analysis was calculated using SPSS v.22 (SPSS Inc., Chicago, IL, USA). The study was approved by the local ethics committee of the Technical University Munich, Klinikum rechts der Isar (File number: 104/18S). Analysis of data acquired during daily ENT examinations was procured from medical hardcopy and electronic file records and then pseudonymized. No further patient agreement was required.

## 3. Results

Our patient collective consisted of 171 consecutively treated patients who underwent L(P)E. All patients underwent tracheoesophageal shunt operation during the L(P)E. Sixty-two patients suffered from TESI that has to be handled with in-hospital procedure, while 109 patients did not. When the patient collective was stratified into those with TESI and those without, no significant difference in gender, age, tumor localization, T, R, or M status was noted (Table 1). The mean age at tumor diagnosis was 59 years in the group of patients with TESI, and 62 years in the group without TESI (*p* = 0.07; Table 1). In both groups, the vast majority of patients (94% vs. 89%; *p* = 0.3; Table 1) were male. Reflecting the indication for L(P)E, both groups demonstrated locally advanced disease (T3/4 status) in more than two-third of cases (*p* = 0.32; Table 1). Interestingly, patients without TESI were more often nodal positive when compared with their TESI-positive counterparts (*p* = 0.001; Table 1). More than 90 percent of patients showed M0 status at the time of diagnosis and showed tumor free margins (Table 1). However, a significant difference was found between the type of therapy (surgery alone vs. surgery with adjuvant radiotherapy vs. surgery with adjuvant chemoradiotherapy) (Table 1; *p* < 0.004). Patients who had surgery alone were less likely to have TESI, as compared to patients with an adjuvant radiotherapy or chemoradiotherapy (Table 1).

A large proportion of L(P)E patients developed the need for an in-hospital treatment of TESI. The median time from surgery to the first episode of TESI was 32 months, with a mean of 47 ± 46 months (Table 2). After initial treatment, 58% of all patients treated on the ward had recurrent TESI. Seventy-nine percent of patients received a non-surgical treatment, while 18% received a transcervical closure of the tracheoesophageal shunt, including major pectoral flap reconstruction. Only 3% of patients received transluminal surgery. Of the 62 patients treated on the ward, 41 patients (65%) underwent successful treatment during their stay. The average duration of time spent in the hospital was 7 days, with a median of 14 ± 22 days (Table 2).

In our study, we categorized the distance of the tracheoesophageal shunt and manubrium in two groups, ≤1.5 cm vs. >1.5 cm. There were striking differences between the groups. TESI-negative L(P)E patients showed a tracheoesophageal shunt-manubrium-distance >1.5 cm in 63% of cases, while tracheoesophageal shunt-manubrium-distance >1.5 cm was achieved in 37% of TESI-positive individuals, only (Table 3, *p* < 0.001). The median distance was 1.21 cm in TESI patients and 1.90 cm in TESI-negative patients, respectively (Table 3).

The median and mean event free survival was 66 months [95% CI: 50; 82] and 77 [95% CI: 63; 91]. Forward selected cox regression including T, N status, primary therapy and categorized distance of the tracheoesophageal shunt and manubrium (≤1.5 cm vs. >1.5 cm) as event modifying parameter revealed that a distance of ≤1.5 cm was associated with an 2.1-fold increased risk in developing hospital required TESI treatment (*p* = 0.01). Interestingly, T status, N status, and primary therapy did not influence event free survival in this cohort.

## 4. Discussion

Voice rehabilitation following L(P)E is one of the most important factors for determining post-operative quality of life. With the inception of the tracheoesophageal shunt and voice prosthesis, patients were able to produce high quality tracheoesophageal voice, and were less likely to be socially stigmatized. Tracheoesophageal voice production with the help of a prosthesis has been associated with a higher quality of life due to excellent voice rehabilitation as viewed by the patient and others. Additionally, patients are also able to more quickly learn how to speak as compared to esophageal speech. Furthermore, patients have a longer duration of phonation as compared to just esophageal speech [14]. However, a common complication is tracheoesophageal shunt insufficiency, which may lead to serious complications, such as aspiration, pneumonia, mediastinitis, and other infections [15,16]. It may be possible that certain surgical procedures can influence the likelihood of shunt insufficiency. However, very little research has been performed to assert and support any mechanism of occurrence. Disorders associated with the voice prosthesis may occur in two pathophysiological manners: either in the form of a prosthetic valve leak, or due to a periprosthetic insufficiency. A prosthetic valve leak may most commonly be attributed to Candida albicans biofilm production. Biofilms leads to destruction, deformation, as well obstruction of the valve, which causes intra-shuntal insufficiency [17,18]. Periprosthetic shunt insufficiency is due to tissue atrophy adjacent to the prosthetic valve. Two forms of periprosthetic insufficiency occur: they may be described as a dilated necrotic shunt insufficiency or an infected necrotic shunt insufficiency. The infected necrotic shunt insufficiency occurs when the tissue between the prosthetic flanges becomes infected. The inflamed tissue becomes edematous and grows in volume, which cannot be compensated by the prosthetic flanges, leading to further inflammation. Eventually the tissue become necrotic, leading to a periprosthetic shunt insufficiency. The dilated shunt insufficiency occurs over many years, and is clinically preceded by the continuous need to reduce prosthetic size. In this case a gradual tissue necrosis develops [19]. Authors identified systemic diseases, such as diabetes mellitus, being associated with shunt insufficiency. Diabetes mellitus may lead to a fundamental change in tissue nourishment: blood vessels become less permeable and intercellular milieu is changed due to long-standing increased sugar levels [20]. A further risk factor for shunt insufficiency is gastro-esophageal-reflux-disease (GERD). Following L(P)E patients have, largely due to the removal of the vocal cords, which provide considerable protection against reflux, a much higher likelihood of developing gastroesophageal reflux. Multiple studies have shown that GERD is a prominent problem in patients following L(P)E. In patients with a tracheoesophageal shunt, reflux is strongly associated with shunt insufficiency. It causes a chronic inflammation of the periprosthetic tissue, and may also cause the growth of granulation tissue which can additionally displace the prosthesis.

In agreement with current literature, in the current study the median timeframe from surgery to the first tracheoesophageal shunt insufficiency in our cohort was 32 months. This indicates that TESI is not an acute post-operative complication, but rather a long-term complication that presents multiple years after surgery and is most likely due to atrophic pathophysiology. In our collective 58% of patients had chronic TESI despite treatment, which is comparable to the literature [21]. The most common approach towards treating shunt insufficiency is a conservative, non-surgical treatment. This amounted to removal of the voice prosthesis, insertion of a cuffed tracheostomy tube, nil per os and feeding via a nasogastric feeding tube. If necessary, low dose prednisolone is administered to reduce peri-shunt inflammation. A conservative approach is considered by most researchers to be the most effective form of treatment. Most surgeons believe that a more caudally located shunt is more likely to develop a shunt insufficiency. The “ideal” location for a tracheoesophageal shunt according to most ENT surgeons is the roof of the endoluminal trachea. Principally, a more cranial location for the shunt provides better protection from gastroesophageal reflux. Additionally, the degree of pressure present in the upper trachea is lower, and could thereby lower the likelihood of an insufficiency due to negative pressure related shunt insufficiency [22,23]. Therefore, the current study exclusively included patients who received tracheoesophageal shunts during primary L(P)E hypothesizing that the distance of the voice prosthesis to the cranial ridge of the manubrium is a crucial factor inducing TESI. This study demonstrated that caudal shunt positioning (≤1.5 cm) was associated with a 2.1-fold increased risk in developing TESI.

An important consideration for shunt insufficiency is an adjuvant radiotherapy. Many researchers believe that radiotherapy leads to tissue metaplasia: vital tissue atrophies and is replaces by scar tissue [24,25]. However, this subject remains controversial, with conflicting results [26]. A study which retrospectively identified 145 patients who received a primary or secondary tracheoesophageal shunt found that no association could be found implicating radiotherapy with shunt insufficiency [27]. The authors found no difference in the complication rate between patients who received no radiotherapy, neoadjuvant radiotherapy, or adjuvant radiotherapy after four years of follow-up. In agreement with recent literature, the forward selected Cox regression in our study failed to demonstrate shunt insufficiency modifying potential with respect to different treatment modalities.

Another potentially implicating factor includes the size of the initial tumor and the extent of the resection during the laryngectomy or L(P)E. Some surgeons believe that larger resection forces surgeons to select a more caudal shunt puncture site, and that more caudally located shunts are more likely to be insufficient. This is an assertion that was supported in the aforementioned study by Gitomer et al. An additional putative risk factor is the metastatic lymph node status, which it is presumed is associated with a higher likelihood for chemoradiotherapy as well as invasive surgery. However, our study found no significant differences concerning gender, age at diagnosis, as well as T, R, and M staging and a risk of shunt insufficiency. Forward selected Cox regression excluded T and N status as being associated with shunt insufficiency. Noteworthy, increased T status can result in deep resection borders and therefore increased distance of the tracheoesophageal shunt and manubrium that we identify as being responsible for the occurrence of shunt insufficiency. Subsequently, secondary puncture after completed wound healing should be performed in order to achieve a higher positioning of voice prosthesis. Accordingly, previous studies have described differences in complication rates between primary and secondary tracheoesophageal puncture [24,28,29].

The positioning of the voice prosthesis is highly associated with TESI. While some decrease of the shunt will occur during wound healing, we have to assume that caudally positioning of the shunts during primary surgical procedure is crucial to induce TESI and therefore, secondary shunt surgery after wound healing should be performed after extensive L(P)E with larger caudal resection margins. However, due to the retrospective nature of the study multivariate analysis of other risk factors, including GERD, radiotherapeutic contouring and severe internistic comorbidity is delusive at this point and has to be included in future prospective trials.

Shunts with a decreased distance to the manubrium sterni were associated with an increased risk of developing an insufficiency. We recommend that a secondary shunt operation be performed if an extensive L(P)E is undertaken. A wider margin between the manubrium sterni and TES lowers the risk of a TESI.

## 5. Conclusions

The tracheoesophageal puncture is commonly performed in patients who have received a L(P)E. TESI is a common problem, and can lead to serious complications. Our study highlights that TESI is a long-term complication of L(P)E associated with shunt positioning during primary surgical procedure. Tracheoesophageal shunts which were more caudally located were associated with an increased risk of developing an insufficiency. We recommend performing a secondary shunt operation when an extensive L(P)E is undertaken, as this would allow a wider margin between the manubrium sterni and the TES, hereby lowering the risk of a TESI.

## Figures and Tables

**Table 1:** Epidemiological data of analyzed collective. TESI = Transesophageal shunt insufficiency.

	TESI Pos.	TESI Neg.	*p*-Value
n	62	109	
Age at tumor diagnosis (years)			0.07
Median	60	62	
Mean ± SD	59 ± 12	62 ± 10	
Sex, n (%)			0.30
Male	58 (94)	97 (89)	
Female	4 (6)	12 (11)	
Diagnosis, n (%)			0.18
Laryngeal carcinoma	41 (66)	67 (62)	
Hypopharyngeal carcinoma	21 (44)	36 (33)	
Oropharyngeal carcinoma	0	4 (4)	
Trauma	0	2 (2)	
Therapy, n (%)			0.004
Surgery	5 (8)	31 (28)	
Surgery + RT	37 (60)	53 (48)	
Surgery + CRT	20 (32)	25 (23)	
T status, n (%)			0.32
T1	0	6 (5)	
T2	20 (32)	27 (25)	
T3	20 (32)	27 (25)	
T4	22 (36)	47 (43)	
N status, n (%)			0.001
N0	39 (63)	56 (51)	
N1	7 (11)	13 (12)	
N2a	12 (19)	7 (6)	
N2b	4 (7)	17 (16)	
N2c	0	12 (11)	
N3	0	2 (2)	
M status, n (%)			0.16
M0	62 (100)	105 (96)	
M1	0	2 (4)	
R status, n (%)			0.53
R0	60 (98)	101 (93)	
R1	2 (2)	6 (2)	
R2	0	0	

**Table 2:** Hospital required treatment of shunt insufficiency. TESI = Transesophageal shunt insufficiency.

	TESI
n	62
Timeframe tumor surgery—insufficiency (months)	
Median	32
Mean ± SD	47 ± 46
Recurrent insufficiency, n (%)	36 (58)
Insufficiency therapy, n (%)	
Conservative	49 (79)
Transluminal surgery	2 (3)
Transcervical surgery incl. major pectoral flap	11 (18)
Treatment succes, n (%)	41 (65)
Hospitalization time (days)	
Median	7
Mean ± SD	14 ± 22

**Table 3:** Positioning of voice prosthesis. TESI = Transesophageal shunt insufficiency.

	TESI Pos.	TESI Neg.	*p*-Value
n	62	109	
Distance Voice prothesis—manubrium (cm)			
>1.5 cm, n (%)	23 (37)	69 (63)	<0.001
Median	1.21	1.90	
Mean ± SD	1.66 ± 1.14	1.83 ± 1.36	

## Data Availability

Study data is unavailable due to privacy or ethical restrictions.
